# ADAMTS-1, a multifunctional proteinase, in the uterus of both estrous cycle rats and ovariectomized rats can be regulated via hormones

**DOI:** 10.1007/s00418-025-02382-5

**Published:** 2025-06-03

**Authors:** Tuba Parlak Ak, Mine Yaman, Ali Bayrakdar, Ozgur Bulmus

**Affiliations:** 1 Department of Nutrition and Dietetics, Faculty of Health Sciences, Munzur University, Tunceli, 62000 Turkey; 2 Department of Histology and Embryology, Faculty of Veterinary Medicine, Firat University, Elazig, 23119 Turkey; 3 Department of Histology and Embryology, Faculty of Veterinary Medicine, Balikesir University, Balikesir, 10000 Turkey; 4 Department of Physiology, Faculty of Medicine, Balikesir University, Balikesir, 10000 Turkey

**Keywords:** ADAMTS-1, Estrous cycle, Ovariectomy, Rat uterus

## Abstract

Remodeling of the extracellular matrix (ECM) throughout the estrous cycle is one of the most striking features of the uterus. A disintegrin and metalloprotease with thrombospondin type I motifs (ADAMTS-1) is a metalloproteinase responsible for the degradation of some proteoglycans, which are ECM components. In this study, ADAMTS-1 distribution was analyzed in the uterus of ovariectomized rats administered 17β-estradiol (E2) and progesterone (P4) and in the uterus at different estrous stages. Ovariectomized (OVX) rats were subjected to single and combined E2 (0.2 mg/kg) and P4 (10 mg/kg) hormone replacement therapies. E2 was administered for 3 consecutive days, followed by E2, P4, or E2 + P4 for 4 consecutive days. The serum level of E2 decreased from the proestrus phase to the diestrus phase, but that of P4 was the highest in the estrus phase. During the estrus phase, the serum level of luteinizing hormone (LH) was the lowest and that of follicle-stimulating hormone (FSH) was the highest. P4 level increased significantly in the OVX + P4 and OVX + E2 + P4 groups compared with the OVX group. The serum levels of LH and FSH decreased in the OVX + E2 and OVX + P4 groups compared with the OVX group, and were the lowest in the OVX + E2 + P4 group. ADAMTS-1 immunoreactivity in luminal, glandular, and stromal cells of the uterus decreased from proestrus to diestrus. When immunoreactivity in hormone replacement groups was compared, weak immunoreactivity was observed in the OVX group. ADAMTS-1 immunoreactivity gradually increased in OVX + P4 and OVX + E2 groups, and was particularly notable in luminal, glandular, and stromal cells in the OVX + E2 + P4 group. ADAMTS-1 distribution was affected by the estrous cycle process and hormone replacement therapy in the OVX procedure.

## Introduction

The a disintegrin and metalloprotease with thrombospondin motifs (ADAMTS) proteases belong to a family of 19 metalloproteinases that play major roles in organ development and tissue homeostasis by regulating extracellular matrix (ECM) formation, remodeling, and homeostatic adaptation (Rose et al. 2021). The metalloproteinase family is composed of molecular structures such as a disintegrin-like and metalloproteinase (ADAM) proteases, matrix metalloproteinases (MMPs), and ADAMTS (Hernández-Delgado et al. 2023). One distinguished member of this family is ADAMTS-1, a well-known ECM protease and the first described member of the ADAMTS family of metalloproteases (Silva et al. 2023). ECM remodeling is an important physiological process related to reproductive capacity in females. This restructuring of the ECM has been associated with the reproductive processes of folliculogenesis, ovulation, implantation, and placentation in females. In addition, the degradation of the ECM in the zona pellucida is an important physiological process for oocyte fertilization. Different matrix metalloproteinases function in the degradation of ECM (Hernández-Delgado et al. 2023).

The reproductive cycle in rodents is regulated by recurring morphophysiological and hormonal changes in these organs (Donner and Lowry 2013). In rats, 17β-estradiol (E2) stimulates epithelial cell proliferation and synthesis of progesterone receptors (PRs). Progesterone (P4) inhibits epithelial cell proliferation while stimulating the proliferation of stromal cells that initiate decidualization (Salgado et al. 2011). The combined effect of E2 and P4 regulates the estrous cycle and prepares the endometrium for implantation. Normal changes in the levels of these ovarian steroid hormones result in cellular differences in the uterine tissue and remodeling of ECM molecules (Salgado et al. 2011). In mice, the deposition of collagen and proteoglycan in the uterine tissue varies during the estrous cycle and early pregnancy (Salgado et al. 2013). In the uterus of ovariectomized (OVX) mice receiving hormone replacement therapy, the expression and deposition of some proteoglycans are modulated differently with the effect of E2 and P4 (Salgado et al. 2009, 2011). In addition, a molecular study indicated that E2 induces early growth response 1 (EGR1) to mediate its effects on the uterine epithelium by controlling P4 receptor signaling for successful implantation. In particular, it has been reported that EGR1 also identifies ADAMTS1 metalloprotease as a new target gene (Park et al. 2021).

Some ECM components expressed during the peri-implantation period are considered to be responsible for the impaired ovulation and implantation of mice with knocked-out ADAMTS-1 genes (Shindo et al. 2000; San Martin et al. 2004). ADAMTS-1, which is involved in the degradation of these proteoglycans, is known to be a prominent component of the uterus–placental environment owing to its potential role in endometrial functions, and its expression in the menstrual cycle is stimulated by gonadal steroids such as P4, E2, and androgens (Demircan et al. 2014).

In this context, the role of ADAMTS-1 in the normal estrous cycle and ovulation, and its ability to regulate endometrial functions, may be possible through cooperation with gonadal hormones. The distribution of ADAMTS-1, which regulates endometrial functions in the uterus, owing to these hormonal changes is therefore suitable for investigation. Whether E2 and P4 potentially regulate the distribution of ADAMTS-1 in the uterus emphasizes the importance of the study. The aim of our study is to examine the immunohistochemical distribution of ADAMTS-1 in the uterine tissue of OVX rats administered E2 and P4 and in the uterine tissues of rats at different estrous cycle stages.

## Material and methods

### Animals and experimental design

**Fig. 1:**
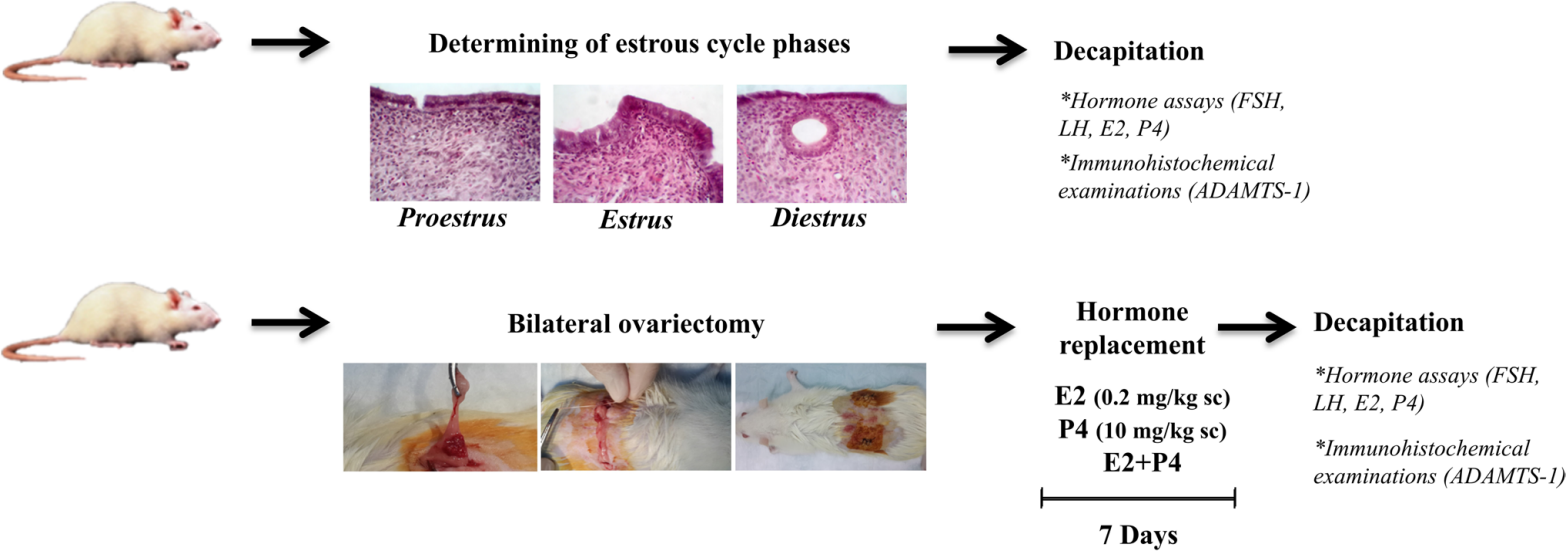
A flow diagram of the experimental process

Forty-eight female Wistar albino rats (3–4 months old) were acquired from Firat University Experimental Research Center (Elazig, Turkey). The animals were provided with optimal situation (50–60% humidity, 22 °C ± 3 °C temperature, optional feed and water, 12 h light/dark cycle) throughout the experimental procedures. This study was approved by the local ethics committee of Fırat University in Elazig (Ethic no.: 13.02.2019–2019/03–31-2019/20). The phases of the estrous cycle were determined through the vaginal smear method, and animals with three consecutive regular cycles in the follow-up period were included in the study (Risvanli et al. 2003). Since the metestrus phase in female albino rats is quite short (6–8 h) compared with other phases, this phase was not included in our study (Sikora et al. 2021). OVX was performed on rats under xylazine (10 mg/kg, intramuscularly [im]) and ketamine (90 mg/kg, im) anesthesia by reaching the ovary with bilateral incisions made from the dorsal area. The protocol of hormone replacement therapy to be administered with E2 (17β-estradiol; Sigma-Aldrich Chemical Co., USA) and P4 (4-pregnene-3,20-dione; Sigma-Aldrich) was adapted from previous studies conducted by Salgado et al. (2009; 2011) (E2 was administered for 3 consecutive days, after 2 days of rest followed by E2, P4, or E2 + P4 for 4 consecutive days). The experimental period was determined as 24 days. A flow diagram of the experimental process is depicted in Fig. 1.

The rats were divided into a total of eight groups (*n* = 6): the different estrous cycle groups: proestrus (group I), estrus (group II) and diestrus (group III); the hormone replacement therapy groups: sham (group IV)—skin–peritoneal incision was made but cycle monitoring was not performed; OVX (group V)—the rats underwent OVX; OVX + E2 (group VI)—15 days after OVX, the rats were administered with 0.2 mg/kg E2 (sc, diluted in sesame oil) for 3 consecutive days, and after 2 days of rest, E2 was administered at the same dose and through the same route for 4 consecutive days; OVX + P4 (group VII)—15 days after OVX, the rats were administered with 0.2 mg/kg E2 (sc, diluted in sesame oil) for 3 consecutive days, and after 2 days of rest, 10 mg/kg P4 diluted in distilled water was administered in the same way for 4 consecutive days; OVX + E2 + P4 (group VIII)—after the same pretreatment and rest periods as those ensured in the previous groups, this group received both E2 (0.2 mg/kg) and P4 (10 mg/kg) in the same way for 4 consecutive days.

At the end of the experiment, all rats were sacrificed under anesthesia then blood specimens and uterine tissues were taken and evaluated for biochemical and immunohistochemical examinations. The serum collected via the centrifugation of the blood samples (5 min, 4000 × g,  4 °C) was stored at −20 °C for subsequent hormone analyses. The uterine tissues were fixed in a formalin solution (10%) for immunohistochemical assessments. Embedding fixed tissues in paraffin was a standard procedure.

### Hormone assays

The serum follicle-stimulating hormone (FSH) (E-EL-R0391; Elabscience, USA), luteinizing hormone (LH) (ENZ-KIT107; Enzo Life Sciences, Switzerland), E2 (ADI-900–008; Enzo Life Sciences, Switzerland), and P4 (ADI-900–011; Enzo Life Sciences, Switzerland) levels were specified by commercial enzyme linked immunosorbent assay (ELISA) kit for rats and studied in accordance with the specified procedures. The optical density was read at 450 nm for FSH and LH plates, and at 405 nm for E2 and P4 plates in a plate reader. Sensitivity of the assays was 1.88 ng/mL for FSH, 5.2 mIU/mL for LH, 28.5 pg/ml for E2, and 8.57 pg/ml for P4. All processes were applied according to Ulker et al. (2020).

### Immunohistochemistry

Immunohistochemical staining in the tissues was achieved by the avidin–biotin–peroxidase complex method (Kahramanogulları et al. 2024). After deparaffinization and rehydration, sections were heated in a microwave oven in citrate buffer (pH 6.0) for 20 min and washed with phosphate-buffered saline (PBS). Sections were incubated with Ultra V block for 5 min at room temperature and then incubated with rabbit polyclonal anti-ADAMTS-1 primary antibody (1/300; ab39194; Abcam, Cambridge, UK) in a humidified chamber for 1 h at 37 °C. After rinsing in PBS, sections were incubated with biotinylated goat anti-polyvalent and then with streptavidin peroxidase for 30 min at room temperature. Staining was completed after the substrate was incubated with a 3,3′-diaminobenzidine tetrahydrochloride (DAB) chromogen (Thermo Scientific, UltraVision Detection System Anti-Polyvalent, HRP/DAB) for 5–15 min, and the sections were washed with distilled water for 5 min. Background staining was performed with hematoxylin. The sections were examined at 20× magnification using a Zeiss Axiolab 5 microscope (Carl Zeiss Ltd.) equipped with an Axiocam 208 color digital camera with Zeiss ZEN 3.5 blue edition software. Cell counts were made by obtaining images from five different random microscope fields from each sample. The image was subjected to a calculation of the area in mm^2^, as well as the intensity and average of DAB ranging from 0 (black) to 255 (total white). Since multiple images were taken from the same slide, the average for each was calculated by formulating the histoscore (Lessey and Savaris 2013).

### Statistical analysis

SPSS 21.0 (SPSS Inc., Chicago, IL, USA) software was preferred. For the statistical analysis of parameters with normally distributed data, one-way analysis of variance (ANOVA) and the post hoc Tukey test were used. The data were presented as means ± standard deviation. Statistical significance was set at *P* < 0.05.

## Results

### Hormone measurements

**Fig. 2:**
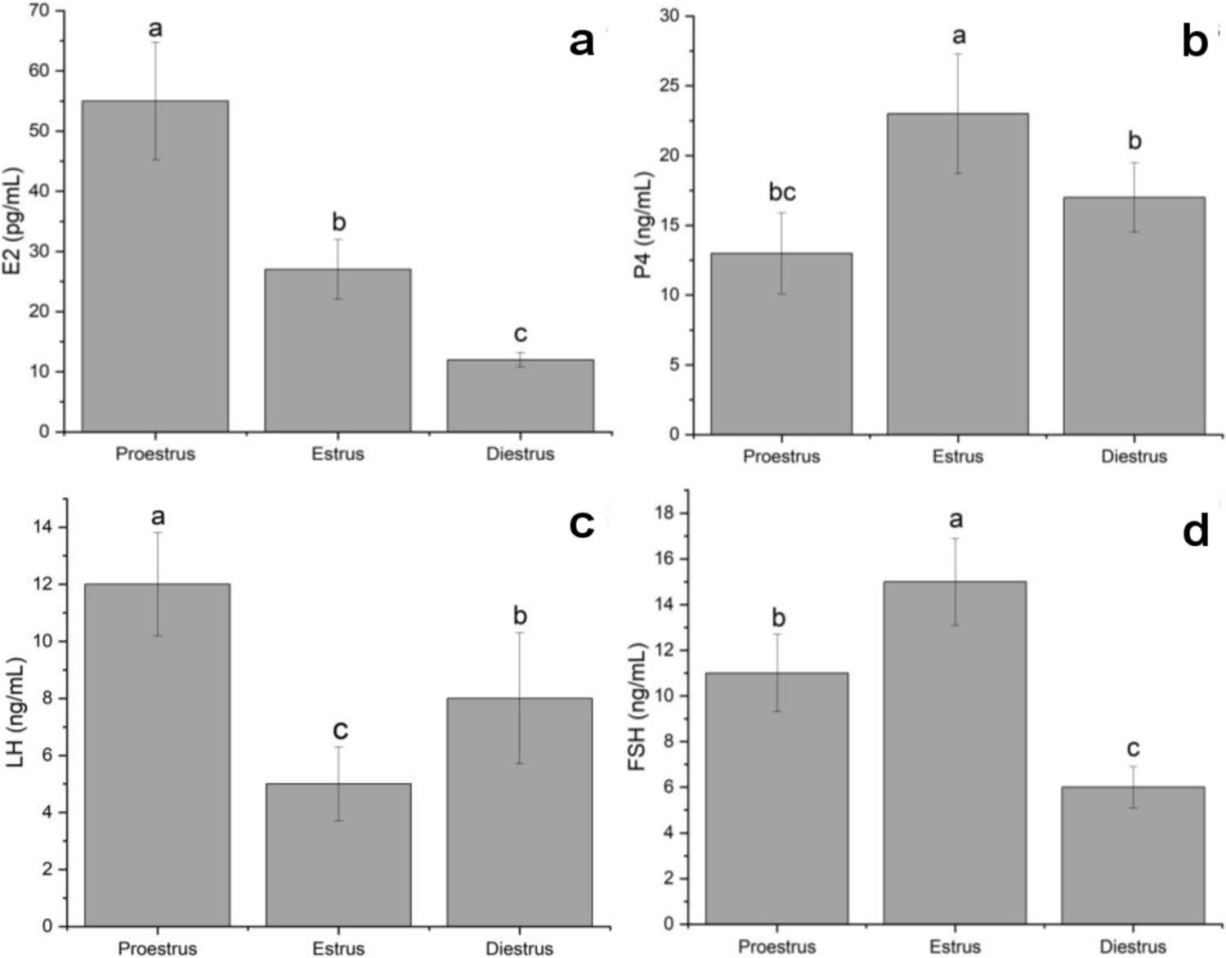
Serum E2 (**a**), P4 (**b**), LH (**c**), and FSH (**d**) levels during estrous cycle stages. Data are given as the mean ± standard deviation for each group (P < 0.05). ^a,b,c^ different letter combinations denote significant differences, *P* < 0.05. *E2* 17β-estradiol, *P4* progesterone, *LH* luteinizing hormone, *FSH* follicle stimulating hormone

The hormone levels determined for the estrous cycle phases are shown in Fig. 2. It was noted that the serum level of E2 was highest in the proestrus phase compared with the levels in the estrus and diestrus phases (55 ± 9.8 pg/mL, *P* < 0.05). The E2 level tended to decrease in the estrus phase (27 ± 5.0 pg/mL, *P* < 0.05) and reached the lowest level in the diestrus phase (12 ± 1.2 pg/mL, *P* < 0.05) (Fig. 2a). It was determined that the serum level of P4 was highest in the estrus phase compared with the levels in the proestrus and diestrus phases (23 ± 4.3 ng/mL, *P* < 0.05). P4 levels tended to decrease in the diestrus phase (17 ± 2.5 ng/mL) and reached the lowest level in the proestrus phase (13 ± 2.9 ng/mL, *P* < 0.05) (Fig. 2b). Serum LH levels were highest in the proestrus phase (12 ± 1.8 ng/mL, *P* < 0.05) and lowest in the estrus phase (5 ± 1.3 ng/mL, *P* < 0.05) (Fig. 2c). Serum FSH levels were highest in the estrus phase (15 ± 1.9 ng/mL, *P* < 0.05) and lowest in the diestrus phase (6 ± 0.9 ng/mL, *P* < 0.05) (Fig. 2d).

**Fig. 3:**
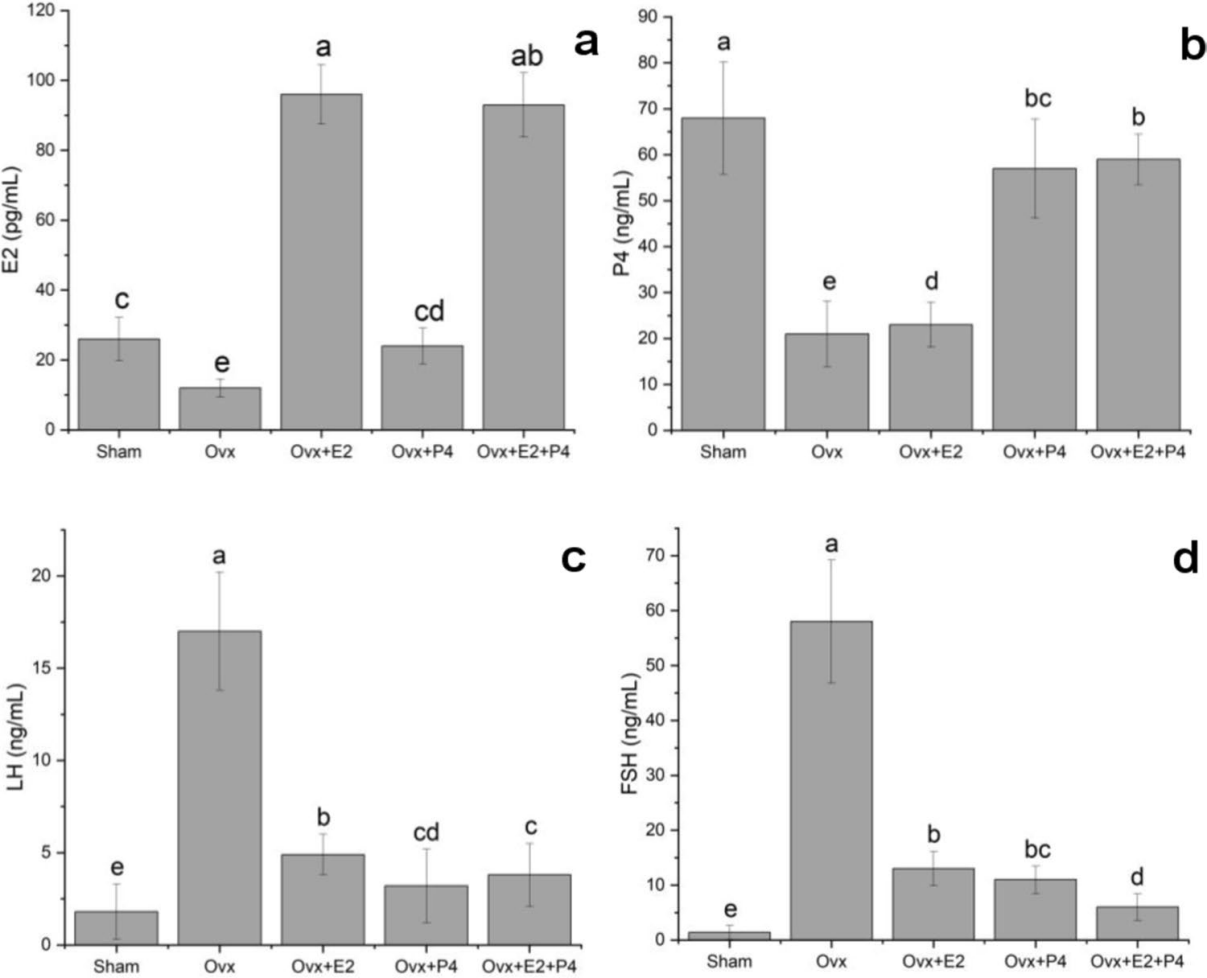
Serum E2 (**a**), P4 (**b**), LH (**c**), and FSH (**d**) levels in OVX and hormone replacement groups. Data are given as the mean ± standard deviation for each group (*P* < 0.05). ^a,b,c,d,e^ different letter combinations denote significant differences; *P* < 0.05. *E2* 17β-estradiol, *P4* progesterone, *LH* luteinizing hormone, *FSH* follicle stimulating hormone

The hormone levels of rats after OVX and hormone replacement therapy are presented in Fig. 3. The serum level of E2 in the OVX group showed a significant decrease compared with that in the sham group (12 ± 2.6 pg/mL, *P* < 0.05). A significant increase was observed in E2 levels in the OVX + E2 and OVX + E2 + P4 groups compared with the OVX group (96 ± 8.5 pg/mL and 93 ± 9.2 pg/mL, respectively, *P* < 0.05). E2 levels in the OVX + P4 group was similar to only that of the sham group (24 ± 5.1 pg/mL) (Fig. 3a). It was noted that there was a low level of P4 in the OVX group compared with the sham group (21 ± 7.1 ng/mL, *P* < 0.05). P4 levels increased significantly in the OVX + P4 and OVX + E2 + P4 groups compared with those in the OVX group (57 ± 10.8 ng/mL and 59 ± 5.5 ng/mL, respectively, *P* < 0.05). The P4 level in the OVX + E2 group was similar to the OVX group level (23 ± 4.9 ng/mL) (Fig. 3b). Serum LH levels were found to be increased in the OVX group compared with the sham group (*P* < 0.05). Compared with the OVX group, LH levels decreased in all hormone replacement groups (*P* < 0.05); the most significant decrease was observed in the OVX + P4 group (3.2 ± 2.0 ng/mL, *P* < 0.05) (Fig. 3c). Serum FSH level was noted to be increased in the OVX group compared with the sham group (58 ± 11.2 ng/mL, *P* < 0.05). Compared with the OVX group, FSH levels decreased significantly in the OVX + E2 and OVX + P4 groups (13 ± 3.1 ng/mL and 11 ± 2.5 ng/mL, respectively, *P* < 0.05); the most significant decrease was observed in the OVX + E2 + P4 group (6 ± 2.4 ng/mL, *P* < 0.05) (Fig. 3d).

### Immunohistochemical findings

**Fig. 4:**
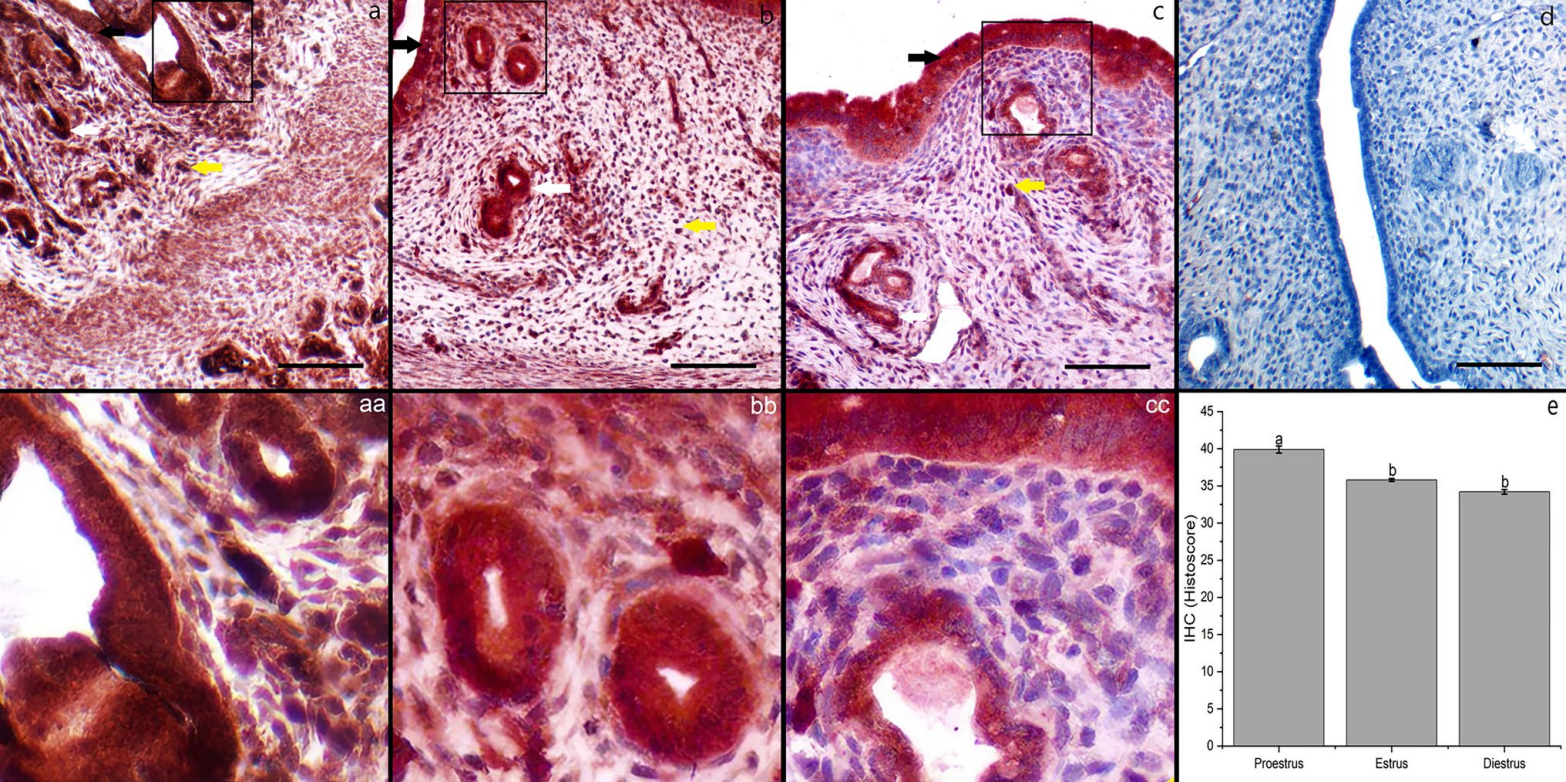
Distribution of ADAMTS-1 immunoreactivity in the uterus during the estrous cycle stages. Proestrus (**a**, **aa**), estrus (**b**, **bb**), diestrus (**c**, **cc**), and negative control (**d**), Histoscore of ADAMTS-1 immunoreactivity (**e**). Representative microphotographs show single letters at 100× and double letters at 200× magnifications. Black arrow: luminal epithelium, white arrow: glandular epithelium, yellow arrow: stromal cells. Data are given as the mean ± standard deviation for each group (*P* < 0.05). ^a,b^ different letter combinations denote significant differences; *P* < 0.05

In the proestrus phase, the immunolocalization of ADAMTS-1 was the most intense and widespread in the luminal and glandular epithelia, and moderately intense in the superficial and deep endometrial stromal cells (Fig. 4a). However, less intense immunolocalization was observed in luminal and glandular epithelia and endometrial stromal cells in the estrus phase than in the proestrus phase (Fig. 4b). In the diestrus phase, ADAMTS-1 immunolocalization was similar to the estrus phase compared with other stages. (Fig. 4c). There was no staining in the negative control (Fig. 4d). The histoscore data confirmed a higher immunoreactivity of ADAMTS-1 in the proestrus phase than in the estrus and diestrus phases (2.51 ± 0.16, *P* < 0.05) (Fig. 4e).

**Fig. 5:**
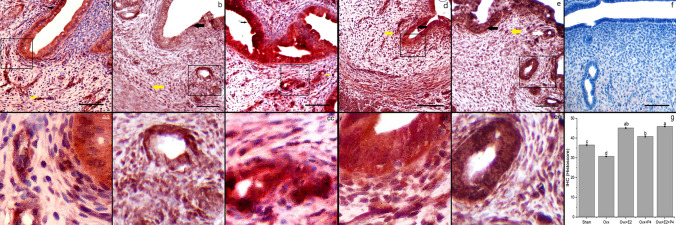
Distribution of ADAMTS-1 immunoreactivity in the uterus following OVX and hormone replacement. Sham (**a**, **aa**), OVX (**b**, **bb**), OVX + E2 (**c**, **cc**), OVX + P4 (**d**, **dd**), OVX + E2 + P4 (**e**, **ee**), and negative control (**f**), Histoscore of ADAMTS-1 immunoreactivity (**g**). Representative microphotographs show single letters at  100× and double letters at  200× magnifications. Black arrow: luminal epithelium, white arrow: glandular epithelium, yellow arrow: stromal cells. Data are given as the mean ± standard deviation for each group (*P* < 0.05). ^a,b,c,d^ different letter combinations denote significant differences; *P* < 0.05

Compared with that in the sham group (Fig. 5a), a weak immunoreactivity of ADAMTS-1 was noted in the OVX group (Fig. 5b). In the OVX + E2 group, moderately intense immunolocalization was observed in the luminal epithelium and endometrial stroma cells compared with the OVX group (Fig. 5c). However, less intense immunolocalization was observed in the luminal epithelium, superficial endometrial stroma cells and inner layer of the myometrium in the OVX + P4 group compared with the OVX + E2 group (Fig. 5d). In the OVX + E2 + P4 group, strong ADAMTS-1 immunolocalization was observed in the luminal and glandular epithelia and the entire endometrial stroma compared with other groups (Fig. 5e). There was no staining in the negative control (Fig. 5f). The histoscore data confirmed that the ADAMTS-1 immunoreactivity was the lowest in the OVX group (0.07 ± 0.02, *P* < 0.05) and the highest in the OVX + E2 + P4 group compared with that in the OVX + E2 and OVX + P4 groups (2.50 ± 0.23, *P* < 0.05) (Fig. 5g).

## Discussion

In our study, we evaluated the intensity and prevalence of ADAMTS-1 immunoreactivity in uterine tissues of rats at different stages of the estrous cycle and of OVX rats treated with E2 and P4.

The reproductive cycle often leads to changes in the levels of E2 and P4, which are produced by the ovaries (Foster and Gray 2010). E2 levels are low at the beginning of the diestrus phase but start to increase toward the end of the diestrus phase, reaching the highest level in the proestrus phase, thus triggering ovulation. The proestrus phase is followed by the estrus phase with a sudden decrease in E2 levels, with the levels continuing to decrease. On the other hand, P4 levels remain high until ovulation and start to decrease toward the end of the estrus phase, and continue to decrease at the beginning of the diestrus phase (Foster and Gray 2010). Our results revealed that the serum level of E2 was the highest in the proestrus phase and gradually decreased in the estrus and diestrus phases. The serum level of P4 increased until the estrus phase and started to decrease after the diestrus phase. Davidge et al. reported that E2 levels decreased in the diestrus phase and that E2 treatment did not cause a significant increase in this level in their study comparing sham and OVX groups (Davidge et al. 2001). Conversely, another study reported that P4 levels in the sham group were on average five times higher than E2 levels, and E2 and P4 levels, which decreased with OVX administration, increased with hormone replacement (Marks et al. 2013). Campbell et al. also stated that the serum levels of E2 and P4 decreased in the OVX group compared with the levels in the sham group; a significant increase was noted in these levels in the E2 + P4 group (Campbell et al. 2003). In the current study, it was determined that the E2 and P4 hormone profile data of the sham group were similar to the findings of Marks et al. (Marks et al. 2013), and that this situation may be due to a complex interaction between ovarian hormones. In addition, this study revealed similar results to the changes in E2 and P4 levels determined in studies where OVX and hormone replacement were applied.

The estrous cycle is a recurrent process under the influence of gonadotropin hormones. FSH and LH are gonadotropins; FSH ensures the growth and development of follicles, whereas LH stimulates ovulation and corpus luteum formation (Aritonang et al. 2017). FSH and LH levels increase and are the highest in the proestrus phase but are the lowest in the estrus phase. In the diestrus phase, the FSH level is the lowest but the LH level increases (Marcondes et al. 2002; Ajayi and Akhigbe 2020). Our findings suggest that FSH and LH levels in the phases of the estrous cycle show a trend similar to that reported in the literature. In our study, we determined that these hormone levels, which increased with OVX administration, decreased with E2 and P4 administration and reached the lowest level in the E2 + P4 combination. In similar studies with OVX, it was reported that serum FSH and LH levels increase (Koebele et al. 2019), and these levels were partially regulated with hormone replacement (Rouach et al. 2011).

The menstrual cycle is a process characterized by various structural changes in endometrial ECM (Demircan et al. 2014). During this cycle, complete tissue repair occurs in the proliferative phase with increasing estradiol levels, early stromal decidualization of the endometrium, menstrual disruption, and mucosal thickening. As estradiol and progesterone levels decrease in the late secretory phase, they lead to induction of menstruation, proteolytic ECM degradation, and shedding of the functional layer, thus ensuring the continuation of the cycle (McLaughlin 2022). This process represents a perfect paradigm for ECM remodeling, and changes in the expression of ECM components are also associated with hormonal fluctuations during the cycle phases (Gaide-Chevronnay et al. 2008). Conversely, it was noted that some proteins belonging to ECM components were not observed in the metestrus stage (Wood et al. 2007). The expression of ADAMTS-1, which is responsible for the degradation of proteoglycans such as syndecan and perlecan in the ECM, is promoted by gonadal steroids such as P4, E2, and androgens (Pelufo et al. 2011; Russell et al. 2015).

ADAMTS-1 expression has been detected in the glandular and stromal cells of the human endometrium in vivo and in vitro (Ng et al. 2006). Its localization has been reported in human endometrium at different stages of the menstrual cycle and pregnancy (Namli Kalem et al. 2018). An experimental study found that *ADAMTS-1* mRNA expression was intense in luminal and glandular epithelial cells in the mouse uterus, especially in the E2-dependent proestrus and estrus stages (Kim et al. 2005). Similarly, in another study, it was stated that ADAMTS-1 was also intense in endometrial stromal cells, in addition to its expression in these cells, and that ADAMTS-1 was regulated by the E2-dependent EGR1 transcription factor in the uterus (Park et al. 2021). In our study, we determined that ADAMTS-1 immunolocalization is more intense in luminal and glandular epithelial and endometrial stromal cells in the proestrus phase, where E2 is dominant, compared with the estrus and diestrus phases. ADAMTS-1 localization in the uterus according to the phases of the estrous cycle may be associated with changes in E2 levels.

It has been reported that the presence of ADAMTS-1 in periovulatory follicles of mice is dependent on PRs in response to LH (Robker et al. 2000) and acts on ECM molecules and/or growth factors in the uterus, which is the main target of P4 (Kim et al. 2006). In PR gene knockout mice, it has been shown that ovulation was absent and *ADAMTS-1* mRNA expression was significantly reduced (Namli Kalem et al. 2017). It has also been reported that *ADAMTS-1* mRNA expression is stimulated by P4 (Mishra et al. 2013), which is effective in implantation, fetal development processes, and endometrial functions (McLaughlin 2022). On the contrary, another study stated that ADAMTS-1 expression was regulated by the E2-dependent EGR1 transcription factor in the uterus, and that ADAMTS-1 was absent in EGR1 knockout mice (Park et al. 2021). In addition, it was determined that ADAMTS-1 expression increased after E2 application and immunofluorescence activity was observed especially in glandular, luminal epithelial, and endometrial stromal cells (Park et al. 2021). Kim et al. determined that ADAMTS-1 gene expression was significantly increased by the combination of E2 + P4 compared with P4 administration alone in ovariectomized mice receiving hormone treatment (Kim et al. 2004). Similarly, in this study, E2 application increased the decreased ADAMTS-1 immunoreactivity compared with P4 in OVX rats receiving hormone treatment, and its localization was observed in luminal and endometrial stromal cells. It was therefore determined that the E2 + P4 combination was more effective. It can be said that the presence of ADAMTS-1, which mediates dynamic tissue regeneration in the uterus, and is affected by hormonal changes during the reproductive cycle, is regulated endocrinically.

This study has some limitations. The study findings could not be adequately compared due to the lack of information on the immunolocalization of ADAMTS-1 in the OVX and hormone replacement models, and in the estrous cycle stages. Even with this lack of information, our study is still considered to have important and current aspects. In our study, ADAMTS-1 localization in the uterus was examined only by immunohistochemical techniques. It is thought that the mechanisms by which ADAMTS-1 acts as a regulator in uterine remodeling and the determination of other related ECM components with different methods may be the focus of our future research.

In conclusion, we examined the uterine endometrium of rats in proestrus, estrus, and diestrus stages, as well as ovariectomized rats receiving hormone replacement therapy for ADAMTS-1 immunoreactivity. We found that the distribution of ADAMTS-1 in the uterus is highly sensitive to estrous stage and hormone replacement therapy, and that this immunolocalization is influenced by tissue-specific hormonal changes. ADAMTS-1, which is thought to contribute to tissue remodeling owing to this hormonal regulation, should be considered for its potential to guide future research.

## Data Availability

No datasets were generated or analyzed during the current study.
